# Combustion ion chromatography for extractable organofluorine analysis

**DOI:** 10.1016/j.isci.2021.102968

**Published:** 2021-08-10

**Authors:** Rudolf Aro, Ulrika Eriksson, Anna Kärrman, Iris Reber, Leo W.Y. Yeung

**Affiliations:** 1 Man-Technology-Environment (MTM) Research Centre, School of Science and Technology, Örebro University, 701 82 Örebro, Sweden; 2 Metrohm AG, Ionenstrasse, 9100 Herisau, Switzerland

**Keywords:** Analytical chemistry, Geochemistry methods, Environmental chemistry, Environmental monitoring

## Abstract

Combustion ion chromatography (CIC) has found a role in environmental analytical chemistry for fluorine content analysis. It is used for extractable organofluorine (EOF) analysis to evaluate perfluoroalkyl and polyfluoroalkyl substances (PFASs) and other organofluorine burden. The prevailing assumption has been that all PFASs are incinerated in CIC and matrix components have no impact on this process, but this has not been experimentally evaluated. In this work, the combustion efficiencies of 13 different PFASs were determined (66–110%). A notable difference was observed between calibrating the CIC with inorganic fluorine or organofluorine. Potential interferences from cations and coextracted matrix components from whole blood and surface water samples were evaluated. These observations should be acknowledged when performing EOF analysis using CIC, overlooking either non-100% combustion efficiencies or the differences in calibrating the CIC with inorganic fluorine or organofluorine could lead to underestimating EOF content and through that to misguide policy decisions.

## Highlights

• Combustion efficiencies of PFASs were in the range of 66–110%
• There was difference between calibration with inorganic fluorine and organofluorine
• Matrix had no discernable effect on combustion efficiency

## Graphical abstract

**Figure:**
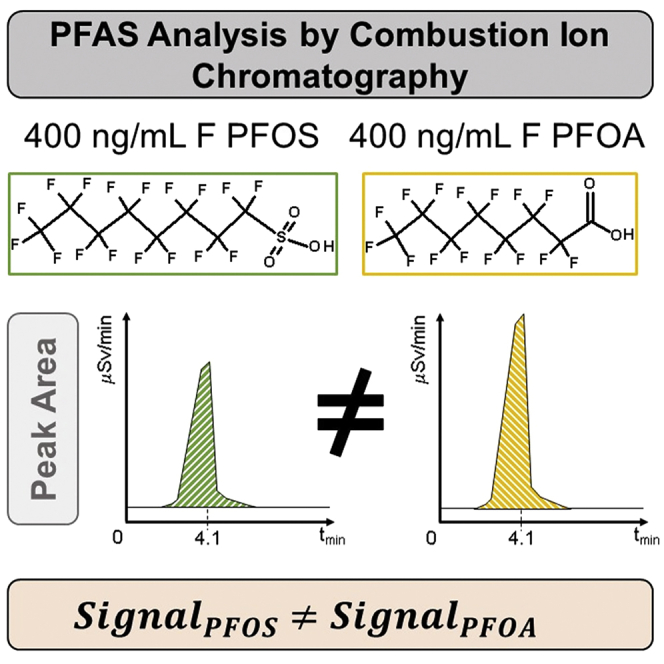

## Introduction

At low concentrations (0.7–1.2 mg/L) in drinking water, fluoride has beneficial effects on human health (United Nations Environment Programme et al., 1984). Excess concentration of fluoride has been linked to negative health effects in plants, insects, and animals (including humans) (Zuo et al., 2018). This stimulated the development of methods to measure fluoride content in water, initially colorimetry (Foster, 1933) and later ion-selective electrodes (ISEs) (US EPA, 2015).

These methods alone were unsuitable for solid samples (e.g., coal), which led to the development of combustion bomb and pyrohydrolysis methods (Thomas and Gluskoter, 1974; Warf et al., 1954) in which a combustion step to release the fluoride is combined with fluoride measurement (e.g., ISE) (Dressler et al., 2003). The combustion methods were later coupled with ion chromatography (IC) and with autosamplers thereafter, allowing the analyst to separate different anions (e.g., fluoride), achieving faster analysis times, and minimizing contamination, using a separate combustion process and offline IC analysis (Saitoh and Oikawa, 1982). The combination of a combustion unit and an attached IC system (combustion ion chromatography [CIC]) has found use in the petroleum and coal industries, where it has been standardized for halogen and sulfur content analysis (e.g., by ASTM International) (ASTM, 2019, 2017). To measure fluoride content, the combustion in CIC is carried out in the presence of water (hydropyrolysis), which is continuously added during the analysis. This shifts the equilibria toward the formation of hydrogen fluoride (can be captured and measured) and away from silicon tetrafluoride (degrades the combustion tube and cannot be measured with IC) (Wagner et al., 2013).

**Figure 1:**
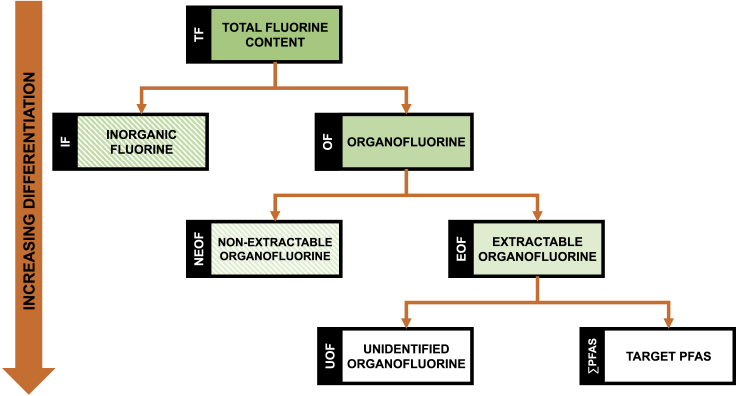
Differentiation of different forms of fluorine

At the turn of the millennia, a new group, perfluoroalkyl and polyfluoroalkyl substances (PFASs), of persistent organic pollutants (POPs) were identified with global environmental distribution (Giesy and Kannan, 2001). Miyake et al recognized the potential in combining CIC analysis with other methods to obtain a more comprehensive picture of the PFAS contamination (Miyake et al., 2007a, 2007b). They combusted the samples at high temperature 1,000–1,050°C, as it had been shown to be necessary to ensure complete degradation of fluorotelomer compounds (Yamada et al., 2005). It has been shown with a few PFASs that their decomposition temperatures can differ significantly (Wang et al., 2015), combustion at 700–900°C results in the formation of volatile PFASs and temperatures above 900°C are necessary to mineralize PFASs (Watanabe et al., 2016). As the CIC method is unable to differentiate between different forms of fluorine, the sample extraction methods had to be modified to remove inorganic fluorine (IF) prior to CIC analysis. The organofluorine (OF) content remaining after extraction, i.e., extractable organofluorine (EOF) content (see Figure 1 for details), was used as an approximation of the PFAS contamination. While natural OF compounds are exceedingly rare (O'Hagan and Harper, 1999), fluorinated agrochemicals and pharmaceuticals are very common (Fujiwara and O'Hagan, 2014; Wang et al., 2014). The difference between the measured EOF content and the fluorine content originating from measured PFASs is labeled “unidentified organofluorine (UOF) content.” Different extraction methods are expected to extract different fractions of OF, making comparison of EOF levels obtained with different extraction methods challenging (Kaiser et al., 2021).

A similar approach is adsorbable organofluorine (AOF) analysis that also makes use of the CIC system, as published by Wagner et al. who brought the AOF methodology to the field of PFAS analysis (Wagner et al., 2013). AOF and EOF differ mainly in that AOF combusts the sorbent with the OF compounds, while EOF combusts the extract from the chosen extraction or clean-up method. Owing to that, AOF and EOF results are not directly comparable. As EOF has been more commonly measured in environmental samples, this article will focus on EOF.

A variety of different techniques in addition to CIC have been use to analyze EOF, for examples, ^19^F nuclear magnetic resonance (NMR), continuum source molecular absorption spectrometry (CS-MAS), and inductively coupled plasma tandem mass spectrometry (ICP-MS/MS) (Jamari et al., 2017; Moody et al., 2001; Qin et al., 2012). Each of these techniques comes with its own limitations: ^19^F NMR does not suffer from interference from inorganic fluorine, but it requires long acquisition times or significant sample preconcentration during sample preparation to overcome its low sensitivity (Koch et al., 2020). While CS-MAS can be coupled with liquid chromatography (LC) to gain more detailed EOF data, the time to pyrolyze the sample in CS-MAS (around 90 s) may result in several OF compounds being analyzed together (Koch et al., 2020). In addition, the combustion efficiencies of different PFASs in CS-MAS are not known. Likewise, ICP-MS/MS can also be coupled with an LC, but ICP-MS/MS analysis of fluoride requires specialized instrumentation and has lower sensitivity than CIC (Koch et al., 2020). A combination of high sensitivity, low cost (compared with, e.g., ICP-MS/MS), and simple operation has resulted in CIC becoming a go-to method for EOF analysis. The use of CIC for fluorine content analysis in environmental samples has become more widely used, and different in-house quality control (QC) routines have been reported (Schultes et al., 2018; Singh et al., 2019; von Abercron et al., 2019). The QC samples could contain a different form of fluorine from the calibration samples (Yeung et al., 2008; Yeung et al., 2009; Yeung and Mabury, 2016; von Abercron et al., 2019), or information regarding QC is lacking (Singh et al., 2019). The use of IF standard solutions to calibrate the system for EOF analysis hinges on the assumed 100% combustion efficiencies of OF compounds. Furthermore, excluding some steps of the instrumental analysis, e.g., by direct injection of the calibration samples onto the ion chromatograph (IC) (Miyake et al., 2007a, 2007b; Yeung et al., 2008), risks overlooking possible sources of error. Using an OF compound for calibration would include the combustion process and help to account for possibly less than 100% combustion efficiency. A selection of studies using CIC is presented in Table 1, and additional details are provided in SI Table S1.

**Table 1:** Selection of studies using CIC to measure EOF

Calibration based on	QC based on	References
Inorganic fluorine	N/A^a^	Miyake et al. (2007a), Miyake et al. (2007b), Loi et al. (2011), Dubocq et al. (2020), Gehrenkemper et al., 2021, Wagner et al. (2013)
	Inorganic fluorine	Schellenberger et al. (2019)
	Organofluorine	Yeung et al. (2008), Yeung et al. (2009), Yeung and Mabury (2016), von Abercron et al. (2019)
Organofluorine	N/A^a^	Singh et al. (2019)
	Organofluorine	Miaz et al. (2020), Schultes et al. (2020b)
	Both	Schultes et al. (2018), Schultes et al. (2020a)

a N/A: no information provided about QC.

To the best of our knowledge, there have been no studies on the combustion efficiencies of different PFAS groups or possible interferences from cations or matrix components in CIC. This study aims to i) improve the quality of fluorine analysis in environmental samples by comparing CIC calibrations using IF and OF, ii) study the combustion efficiencies of different groups of PFAS, and iii) evaluate matrix interferences from surface water and whole blood and how it influences fluorine determination.

## Results and discussion

In this section, we will address the issues of CIC calibration using OF and IF solution, combustion efficiencies of different PFASs, effect of cations on fluorine analysis, effect of matrix components, and possible sources of background fluorine.

### Calibration with OF and IF solutions

Several of the studies listed in Table 1 used a combination of IF and OF samples for calibrating and checking their system (e.g., a NaF calibration curve checked against samples of perfluorobutane sulfonic acid [PFBS] (von Abercron et al., 2019)). However, nearly half of the studies in Table 1 (7 out of 16) did not provide any information regarding QC or whether any samples were used to verify the calibration. Furthermore, none of the studies in Table 1 assessed the analytical performance of their CIC systems for both IF and OF across the whole calibration range.

**Figure 2:**
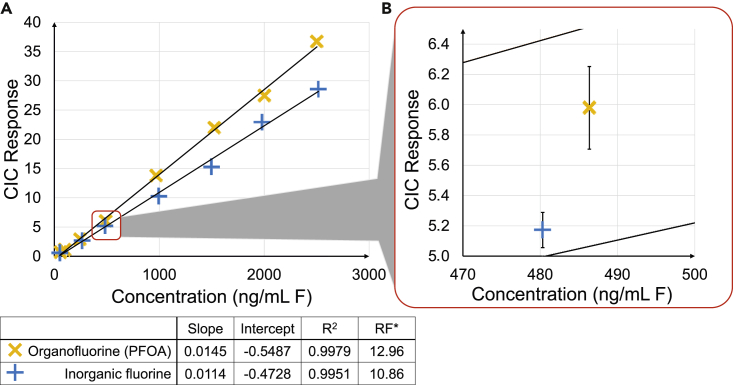
Calibration curves from inorganic fluorine and organofluorine (A) Full calibration range (50–2,500 ng/mL (F). ∗RF – the average response factor, CIC response/concentration (ng/mL (F). (B) Results from triplicate measurements; error bars correspond to standard deviation.

To investigate possible differences between the use of IF and OF as calibration samples, calibration curves between 50 and 2,500 ng/mL F were prepared using methanol (details in STAR Methods section) and analyzed using combustion to ensure all potential sources of error that a sample encounters during its analysis were taken into account. The results from these measurements, i.e., all samples analyzed with combustion, are presented in Figure 2A. The calibration curves were constructed from single measurements owing to long analysis times, and the repeatability of IF and OF calibration samples was estimated by triplicate analysis of one of the calibration samples (one of IF and OF); the respective relative standard deviations (RSDs) of those measurements were 2 and 5% (shown in the enlarged section of Figure 2B).

Both linear calibration curves (without weighting) had correlation coefficients exceeding 0.99, and the RFs differed by 19%, see Figure 2. The RSD of RFs within either calibration curve was below 15%. However, the slope and intercept values for both calibration curves were markedly different. The slope was 28% higher for OF (0.0145 vs 0.0114), and the intercept differed by 16% (OF: −0.5487 and IF: −0.4728).

The results presented in Figure 2 showed that for the method and instrument used in this study, the analytical response for OF and IF can differ by a factor of 0.3. This could lead to either underestimating or overestimating the fluorine content in a sample, depending on which form of fluorine is measured in the sample and which form of fluorine is used for calibration.

For those research groups working primarily with EOF, it may be possible to manage this potential source of bias by calibrating their CIC instrumentation using an OF model compound (e.g. perfluorooctane sulfonic acid [PFOS] or PFOA) or by using QC samples based on OF, preparing them at several concentration levels (e.g., low, medium and high as suggested by the Food and Drug Administration [“Bioanalytical Method Validation Guidance for Industry,” 2018]), and using them to verify the IF-based calibration. An inverse approach could be used for the quantification of IF.

However, both forms of fluorine are present when measuring total fluorine (TF) concentrations, the analysis of a liquid or a solid sample without any sample preparation. If the analytical method has been proven to have the same response to both IF and OF, then either of them could be used for quantification. Should the analytical response differ for the two forms of fluorine, a more careful approach would have to be applied. First, the amount of IF and TF should be determined separately, if possible. This would inform the analyst on which form of fluorine constitutes the majority of TF in the study samples. For example, in blood samples (no extraction), it has been shown that the concentration of IF was an order of magnitude higher than that of OF (Yeung et al., 2009). In such a situation, the calibration for TF analysis should be performed with IF. If the IF and OF concentrations are comparable, the decision should be left to the analyst undertaking the study as he/she would have a better understanding of the samples in question, but the chosen method should be described in detail in any resultant publications or reports.

### Combustion efficiencies of PFASs

There has been only limited work done to determine the combustion efficiencies of different PFASs (Wang et al., 2015), and they are often assumed to be constant, 100%. This is despite the EOF analysis, which relies on this assumption, becoming more widespread and using a variety of analytical techniques in addition to CIC: for example, CS-MAS, fluorine nuclear magnetic resonance (^19^F NMR), and CIC (Koch et al., 2020). Despite CIC being a go-to method for EOF analysis, it is assumed that the combustion efficiencies (conversion of PFASs to HF during hydropyrolysis) for different analytes are the same, such as fluorotelomer phosphate diesters (diPAPs) and PFOS. This is further extrapolated to all of EOF, much of which is often of unknown origin.

**Figure 3:**
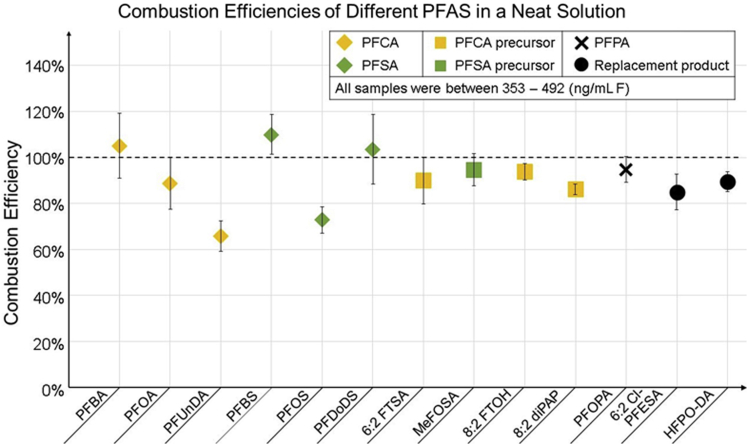
Combustion efficiencies of different PFAS in neat solutions Relative combustion efficiencies of selected PFAS; mean of 3 replicate measurements, error bars correspond to standard deviation.

The results presented in Figure 2 used PFOA as the model compound for the OF calibration. However, environmental samples often contain several PFASs in addition to PFOA. Although this method of calibration should be fit for purpose, data on the combustion efficiencies of different PFASs have not been published for the CIC analysis so far. To this end, a selection of PFAS were analyzed in triplicates (preparation in Method Details). The results from these measurements are presented in Figure 3. The combustion efficiencies for this experiment were obtained by dividing the measured concentration (calculated using a calibration curve prepared from a separate PFOA standard, not the one included in the figure) with the prepared concentration. The mean combustion efficiencies ranged from 66% (perfluoroundecanoic acid [PFUnDA]) to 110% (PFBS). Of the 13 compounds, 3 had combustion efficiencies above 100% (PFBS, 110%, perfluorobutanoic acid [PFBA, 105%], and perfluorododecane sulfonic acid [PFDoDS, 103%]). Four of the compounds had combustion efficiencies between 90 and 100%: *N*-methyl perfluoro-octanesulfonamide (MeFOSA, 95%), perfluorooctylphosphonic acid (PFOPA, 95%), 8:2 fluorotelomer alcohol (8:2 FTOH, 94%), and 6:2 fluorotelomer sulfonic acid (6:2 FTSA, 90%). Furthermore, 4 compounds fell into the next decile: PFOA (89%), hexafluoropropylene oxide dimer acid (HFPO-DA, 89%), 8:2 diPAP (86%), and 6:2 chlorinated polyfluorinated ether sulfonate (6:2 Cl-PFESA, 85%). The lowest combustion efficiencies were observed for PFOS (73%) and PFUnDA (66%). The standards used for these samples were also analyzed for their inorganic fluorine content using direct injection on the IC; no quantifiable amounts of IF were found.

Wang et al. did not identify chain length as something that altered PFSA combustion efficiency (they tested perfluorohexane sulfonic acid [PFHxS] and PFOS) (Wang et al., 2015). This is corroborated by the results from this study, as there did not seem to be a correlation between PFSA chain length and combustion efficiency. At the same time, the combustion efficiencies of perfluoroalkyl carboxylic acids (PFCAs) decreased with increasing chain length (C4 – 105%; C8 – 89%; C11 – 66%). Concurrently, the combustion efficiency of 8:2 diPAP was 86%, a compound with 16 perfluorinated carbons. These results suggest that different functional groups result in different thermal degradation profiles. This has been reported by Wang et al who studied PFOS, PFOA, and perfluorooctanesulfonamide (FOSA) (Wang et al., 2015). The combustion efficiencies of perfluoroalkyl acid’s (PFAA) precursors included in this study had high combustion efficiencies (86–95%), suggesting that CIC does not result in a large underestimation of PFAA precursor content. Likewise, the PFOS and PFOA replacement products had high average combustion efficiency (87%), suggesting CIC's suitability to measure them in environmental samples.

Although the results in Figure 3 show the suitability of CIC for the measurement of 13 different PFAS, the number of potential analytes in environmental samples is several orders of magnitude higher (OECD, 2018). It would not be possible to determine the combustion efficiencies for all of them, but high-purity standards are available for a dozens of PFAS (Wellington Laboratories, 2016). Therefore, work toward a better understanding of their behavior during CIC analysis should be pursued. This should also include fluorinated pharmaceuticals and agrochemicals, as they are often suspected to be part of the unidentified organofluorine fraction (Koch et al., 2020).

### Impact of cations on fluorine analysis

The results presented earlier were obtained by measuring the concentrations of IF and OF in neat solutions without any interfering elements or compounds. However, this might not hold true with environmental samples. In the case of EOF analysis, additional matrix elements may be coextracted during sample preparation, and for TF analysis, the sample is analyzed directly (no sample extraction) (Miyake et al., 2007a; Schultes et al., 2018; Yeung et al., 2009). While TF ensures that no form of fluorine is excluded from the analysis, as the order of magnitude of IF can be several orders of magnitude higher than that of OF (Spaan et al., 2020; Yeung et al., 2008), small changes in OF concentrations cannot be observed in TF measurements. While TF analysis ensures that no form of fluorine is excluded from the analysis, it also increases the risk of interferences. For example, calcium is the fifth most abundant element in the Earth's crust and is also found in water (Hanusa, 2019). Metals such as Ca, Mg, Na, and K can form fluoride salts and could therefore have an influence on the HF formation equilibrium during the combustion process. While the extraction methods may not be optimized for the extraction of cations, this may be counteracted by the very high concentrations of such species in the sample. Moderately, “hard water” as defined by the United States Geological Survey (USGS) has a CaCO_3_ concentration between 61 and 120 mg/L (Briggs and Ficke, 1977), taking 90 mg/L as a starting point; even with a 0.001% recovery, the concentration after extraction would be 900 ng/L.

**Figure 4:**
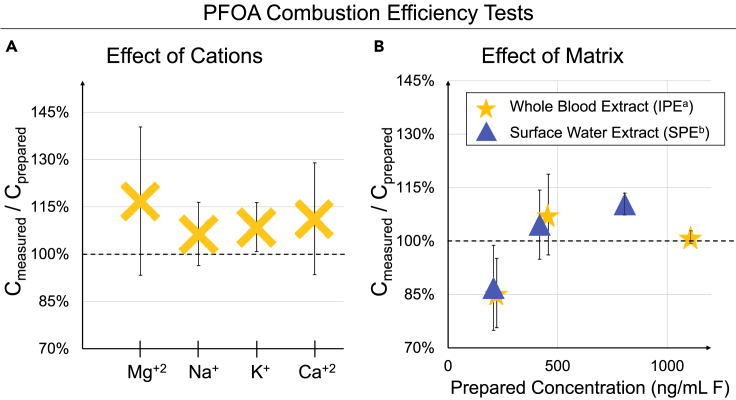
Experiments to determine PFOA combustion efficiency in the presence of matrix components (A) PFOA (500 ng/mL) (F) combustion efficiency in samples spiked with different salts. (B) PFOA combustion efficiency in whole blood and surface water sample extracts. Sample extracts from ^a^IPE: ion-pair extraction; ^b^SPE: solid phase extraction. Each point is a mean of three measurements; error bars correspond to standard deviation.

The potential impact of cations on the hydropyrolysis of PFOA was tested by spiking different salts into standard samples of PFOA in MeOH (see Figure 4A). The mean combustion efficiencies (C_measured_/C_prepared_) for all of the samples in this experiment were in the range of 106–118%. For some of the samples, the RSD was elevated. Therefore, these results should be interpreted cautiously.

The results presented in Figure 4A showed that the presence of different cations at a concentration of ∼230 ng/mL did not have an impact on the combustion efficiency of PFOA in a neat solution. The salt solutions were not prepared at a higher concentration because high levels of alkali metals are destructive to the quartz combustion tube and significantly shorten its lifespan. However, these and other cations could be present at much higher concentrations during TF analysis. Their potential impact on TF analysis could be estimated by spiking the sample with known amounts of OF compounds. On the other hand, the impact of cations on the combustion efficiencies of unknown OF compounds could be estimated by taking a subsample from the study and spiking it with a relevant concentration of salts, but this could result in a shortened life span of the instrumentation and it requires sufficient sample material for such work.

### Impact of matrix components

As OF content analysis using CIC requires the removal of IF during sample preparation, because CIC could not differentiate between OF and IF, the EOF analysis is always performed after extraction. The solid-phase extraction (SPE) method used for surface water extraction was modified with an additional washing step to remove IF (Miyake et al., 2007a). During ion-pair extraction (IPE) of blood samples, the IF is left behind in the aqueous phase as the organic solvent is removed (Miyake et al., 2007b). In both the cases, the sample extraction also serves to remove some of the other compounds or elements that could interfere with CIC analysis. Although matrix-matched calibration is used in bioanalytical methods such as liquid chromatography mass spectrometry (LC-MS) (Kleigrewe et al., 2011), it has not been attempted before for CIC analysis.

Whole blood and surface water samples were extracted using methods described in Method Details, and these sample extracts were spiked with a known amount of PFOA in MeOH at different concentrations (200–1,100 ng/mL F) to test the combustion efficiency of OF in the presence of coextracted matrix components. Both sample extracts were also analyzed without adding PFOA to measure the background EOF concentration. The combustion efficiencies presented in Figure 4B (C_measured_/C_preapred_) were calculated after subtracting the measured EOF concentration in the nonspiked samples; the EOF levels in non-spiked whole-blood extracts were 72 ng/mL F and 70 ng/mL F in surface water samples.

The PFOA combustion efficiencies were between 85 and 110% with both the matrices. The results presented in Figure 4B indicated that the CIC analysis was not affected by the presence of matrix components. This and the results shown in Figure 4A lend credence to the current state of the art that CIC does not require matrix-matched calibration, as the sample is combusted and little if anything of the matrix makes its way to the IC. However, the results in Figure 4 are obtained with PFOA; the results might differ with other PFAS as their combustion efficiencies are not constant (see Figure 3).

In addition, the samples used for the matrix tests were extracted using SPE and IPE (surface water and blood samples, respectively), and using a different extraction method could yield different results as only the PFAS that are extracted can be analyzed further on. Each extraction method captures a different fraction of the PFAS in a sample; for example, the solid-phase extraction-weak anion exchange (SPE-WAX) cartridges can be eluted separately for neutral and anionic PFAS. Thus, when analyzing the anionic fraction for EOF content, it is only the EOF content from anionic compounds. As there are many extraction methods and samples, it would be advisable to use a matrix-matched sample to verify that the coextracted matrix components do not interfere with EOF content analysis using CIC.

The probability of matrix effects is likely to be higher during TF analysis because the whole sample is introduced into the combustion chamber. There, it would be important to test whether the matrix components interfere with CIC analysis with a matrix-matched sample, analyzed as is and after spiking with IF or OF.

### Source of background fluorine

The background fluorine content, as measured by combustion blanks, is often the parameter that determines what concentration can be measured with a high confidence level. While several changes were made to the instrument to lower the background (details in STAR Methods section), a background fluorine signal remained. For example, during the measurements for Figure 4B, the average background signal was equal to 65 ng/mL F in the sample, with an RSD of 17%.

To further investigate this, two components of the analytical procedure were studied in detail: the argon gas and MilliQ water were added to the system during hydropyrolysis. Argon is used to carry HF from the combustion tube into the absorber solution, while water is continuously added into the combustion tube to humidify it and promote HF formation. The rate of their addition was changed in the method parameters, and combustion blank analyses were performed with the modified methods to determine if it had an impact on the combustion blanks.

**Figure 5:**
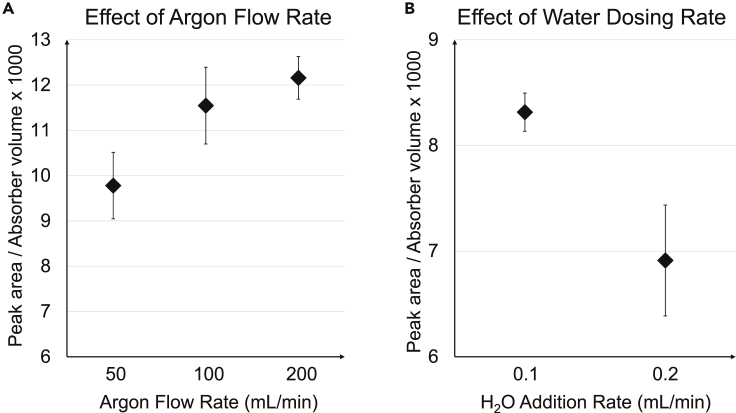
Sources of background fluorine (A) Effect of argon flow rate on combustion blank peak areas. (B) Effect of water dosing rate on combustion blank peak area. All measurements were a mean of 3 replicates. Error bars correspond to standard deviation.

Neither of these variables had a large impact on the background signal (Figure 5). While the peak area increased with higher argon flow rates (Figure 5A), the differences between the different settings were not statistically significant (p < 0.05, 1-way analysis of variance). The results obtained with the lower water dosing rate (0.1 mL/min) appear to be substantially higher (Figure 5B), but the differences were not statistically significant (p < 0.05). A confidence interval of 2 would result in mean values overlapping with each other in both the case studies (effect of argon and water addition).

### Conclusions

The main findings from this work were the different CIC responses to IF and OF and the determination of combustion efficiencies for a selection of PFAS. While some studies using the CIC method have had thorough QC procedures (using both SRM samples of IF and OF), this is not always the case (see Table 1). Gehrenkemper et al. compared high-resolution continuum-source graphite furnace molecular absorption spectrometry (HR-CS-GFMAS) with CIC for quantitative analysis of PFAS contamination in surface water samples and found them to be in good agreement (Gehrenkemper et al., 2021). However, the CIC analysis technique itself remains woefully understudied for the purpose of fluorine content analysis in environmental samples. Because an internal standard is not commonly used in CIC analysis, as is done for example in LC-MS by using mass labeled internal standards, it is not possible to account for potential losses of the analyte during injection, combustion, absorption, or IC analysis. Therefore, it is paramount to better characterize CIC and determine where its limitations lie.

In light of the results of this work that the CIC response is different for OF and IF (see Figure 2), it is crucial to select the appropriate form of fluorine for one's application or verify the method with rigorous QC procedures (e.g., analyzing QC samples with both IF and OF). This should be performed at fluorine concentrations comparable with the study samples. To catch possible interferences and to avoid overestimating the method's capabilities, the calibration and QC samples should be analyzed identically with study samples (i.e., undergo combustion). Regardless of which form of fluorine is used for calibration, the authors should make it abundantly clear to the reader on how the calibration and analysis of study samples were performed. This would ensure that misleading conclusions would not be drawn, should potential sources of bias be identified in the future.

To the best of our knowledge, this is the first work publishing results on the combustion efficiencies of PFAS in CIC analysis. For the 13 compounds studied here, the combustion efficiencies ranged from 66 to 110%, but it remains to be determined for other PFAS and organofluorine compounds (e.g., agrochemicals and pharmaceuticals). While the latter will require further work, the initial tests in this study indicate that the presence of sample matrix for EOF analysis is negligible. However, this would be dependent on the extraction method and potentially of more concern during TF analysis owing to the larger amount of matrix being introduced to the combustion chamber. These considerations are not exclusive to CIC; the combustion efficiencies should be studied for other fluorine measurement methods that also rely on combustion of the OF compounds, such as HR-CS-GFMAS.

Although the current “state of the art” has been sufficient for exploratory work, regulatory bodies have greater demands on accuracy, reproducibility, and robustness. This is of importance because EOF analysis using CIC has great possibilities to be used in monitoring for compliance purposes, i.e., for PFAS-Total.

### Limitations of the study

The results presented in this study are conditional on the instrumentation and analytical methods used; for example, combustion efficiencies could differ with an instrument from another manufacturer or with another method. The example with different matrices serves as a case study and cannot be extrapolated to other extraction methods. This study presents methods on how to assess method suitability for fluorine analysis in environmental samples using combustion ion chromatography; however, it should be evaluated on a case-by-case basis.

## STAR★Methods

### Key resources table

**Table:** 

REAGENT or RESOURCE	SOURCE	IDENTIFIER
**Chemicals, peptides, and recombinant proteins**		

Fluoride standard solution	Merck KGaA	Cat#1.19814.0500
Anion multi-element standard I	Merck KGaA	Cat#1.11437.0500
Perfluorooctanoic acid (>96%)	Aldrich	CAS 335-67-1
Perfluoro-n-butanoic acid (>98%)	Wellington Laboratories	Cat#PFBA
Perfluoro-n-[1,2-13C2]octanoic acid (>98%)	Wellington Laboratories	Cat#M2PFOA
Perfluoro-n-[1,2-13C2]undecanoic acid (>98%)	Wellington Laboratories	M7PFUdA
Potassium perfluoro-1-butanesulfonate (>98%)	Wellington Laboratories	Cat#L-PFBS
Sodium perfluoro-1-[13C8]-octanesulfonate (>98%)	Wellington Laboratories	Cat#M8PFOS
Sodium perfluoro-1-dodecanesulfonate (>98%)	Wellington Laboratories	Cat#L-PFDoS
Sodium 1H,1H,2H,2H-perfluorooctane sulfonate (6:2) (>98%)	Wellington Laboratories	Cat#6:2FTS
N-methylperfluoro-1-octanesulfonamide (>98%)	Wellington Laboratories	Cat#N-MeFOSA-M
2-Perfluorooctyl ethanol (8:2) (>98%)	Wellington Laboratories	Cat#FOET
Sodium bis(1H,1H,2H,2H-perfluorodecyl)phosphate (>98%)	Wellington Laboratories	Cat#8:2diPAP
Perfluorooctylphosphonic acid (>98%)	Wellington Laboratories	Cat#PFOPA
Potassium 9-chlorohexadecafluoro-3-oxanonane-1-sulfonate (>98%)	Wellington Laboratories	Cat#9Cl-PF3ONS
2,3,3,3-Tetrafluoro-2-(1,1,2,2,3,3,3-heptafluoropropoxy)propanoic acid (>98%)	Wellington Laboratories	Cat#HFPO-DA
Methanol (>=99.8%)	Fisher Scientific	Cat#M/4056/17X
Methyl tert-butyl ether (>=99.8%)	Sigma-Aldrich	Cat#34875
Tetrabutyl-ammonium sulfate (>=99.0%)	Sigma-Aldrich	Cat#86868
Oasis weak anion exchange cartridges	Waters Corporation	Cat#186002493
Potassium chloride (reagent grade)	Scharlau	Cat#PO0200
Magnesium chloride (reagent grade)	Scharlau	Cat#MA00360500
Sodium Chloride (reagent grade)	VWR Chemicals	Cat#27810.295
Calcium chloride (reagent grade)	Merck	Cat#1.02382.0500
Argon (6.0)	AGA AB	CAS 7440-37-1
Oxygen (5.0)	AGA AB	CAS 7782-44-7

**Software and algorithms**		

MagIC Net 3.3	Metrohm AG	https://www.metrohm.com/en/support-and-service/software-center/magic-net/

**Other**		

930 Metrohm Combustion IC PP	Metrohm AG	https://www.metrohm.com/en/products/ion-chromatography/930-compact-ic-flex/29309010

### Resource availability

#### Lead contact

Further information and requests for resources and reagents should be directed to and will be fulfilled by the lead contact, Leo Yeung (leo.yeung@oru.se).

#### Materials availability

The study did not generate any unique reagents.

### Method details

#### Chemicals and reagents

Standard reference material (SRM) solutions made from inorganic fluorine “fluoride standard solution” (nr. 1.19814.0500) and “anion multielement standard I” (nr. 1.11437.0500) were purchased from Merck KGaA, Darmstadt, Germany. PFOA in solid form was bought from Aldrich, now part of Sigma-Aldrich (St. Lousi, MO, United States). Standard solutions of PFAS were purchased from Wellington Laboratories (Guelph, Canada). Potassium and magnesium salts (KCl, reagent grade; MgCl_2_ x 6 H_2_O, reagent grade) were purchased from Scharlau (Barcelona, Spain). Sodium chloride (NaCl) was purchased from VWR Chemicals (Radnor, PA, US). Calcium chloride (CaCl_2_ x 2 H_2_O) was purchased from Merck.

Methanol (MeOH) was purchased from Fisher Scientific (Waltham, MA, United States). Water for sample preparation and the CIC (absorber, rinsing, eluent solutions) was purified with a MilliQ system (18.2 MΩ resistance). Argon and oxygen for the CIC were purchased from AGA (Lidingö, Sweden). Methyl tert-butyl ether (MTBE) and tetrabutyl-ammonium sulfate (TBA), used for IPE, were purchased from Sigma-Aldrich. SPE cartridges (6 mL, 150 mg sorbent, 30-μm particle size) with WAX sorbent were purchased from Waters Corporation, Milford, MA, US. GF/F glass fiber filters (150 mm, 0.7 μm) were purchased from Whatman (Chicago, IL, US).

#### Preparation of samples and standards

Standards of IF were prepared from SRM solutions into volumetric flasks and filling them to the mark with MeOH. The IF standards were prepared in MeOH because these standards were also combusted, just like OF standards. This was done to ensure that the different standards are handled as similarly to each other as possible. The SRM solution was pipetted with a calibrated automatic pipette; the mass of this addition was recorded by weighing the flask. To avoid handling very small amounts of SRM solutions, possibly resulting in a large relative error, only volumes exceeding 0.5 mL were pipetted. Calibration samples of OF were prepared from solid PFOA, dissolved in MeOH. Solvent additions were done by weight, and the stock solutions prepared from solid PFOA were added by volume using an automated analytical syringe from SGE (now part of Trajan Scientific and Medical, Ringwood, Australia). Solutions for testing combustion efficiencies of the different PFAS were prepared by weighing the amount of solvent (MeOH) and adding a fixed amount of the PFAS standard solutions from Wellington, using the automated analytical syringe. Salt solutions for testing the impact of cations on OF combustion efficiency were prepared by weight in pure MeOH (CaCl_2_, MgCl_2_) or 90/10 v/v MeOH/H_2_O (NaCl, KCl).

The surface water and whole-blood sample extracts used in this study were extracted using the previously published methods (Yoo et al., 2009; Eriksson et al., 2017) (“ISO 25101:2009,” 2009).

The whole-blood samples were extracted with IPE; in brief, 3.0 mL of whole blood was mixed with 5 mL of MTBE and 2 mL of 0.5 mol/L TBA in water. The samples were shaken and then centrifuged to remove the top layer; the extraction was repeated twice with 3 mL of MTBE. The combined MTBE extracts were evaporated to 0.2 mL under a stream of nitrogen, before being reconstituted to 1.0 mL with MeOH, and evaporated down to 0.5 mL. This method achieves the separation of IF from the sample by the use of the aqueous TBA solution, which dissolves the IF, while OF compounds are extracted into the organic solvent.

The surface water samples were extracted with SPE-WAX cartridges (6 mL, 150 mg sorbent, 30 μm particle size). The cartridges were conditioned with 4 mL of MeOH, 0.1% NH_4_OH, 4 mL MeOH, and 4 mL MilliQ water. This was followed by loading 200 mL of the sample. The cartridges were washed with 20 mL of 0.01% NH_4_OH in water to remove inorganic fluorine, followed by 10 mL of MilliQ water and 4 mL of 25 mmol/L ammonia acetate buffer in water and 4 mL 20% MeOH solution. After drying the cartridges under vacuum for 30 minutes, the cartridges were eluted with 4 mL of 0.1% NH_4_OH in MeOH and evaporated to 0.5 mL.

#### Instrumental analysis and quantification

EOF levels were determined with a CIC system (Figure S1) comprised of a combustion module, an Auto Boat Drive (ABD), a MMS 5000 (autosampler) (Analytik Jena, Germany), a 920 Absorber Module, and a 930 Compact IC Flex ion chromatograph module (Metrohm, Switzerland). An ion-exchange column (Metrosep A Supp 5 - 150/4.0), with carbonate buffer (64 mmol/L sodium carbonate and 20 mmol/L sodium bicarbonate) as the mobile phase, was used for the separation of anions; the absorber solution was water. All samples, including standards, included in this work were analyzed with combustion, unless otherwise stated. Details of the method are in SI 2 Table S2.

The sample extract (100 μL) was injected onto a quartz glass sample boat using the autosampler, and the samples were combusted at 1050 °C, in the presence of argon (6.0, purity; 100 mL/min) and oxygen (5.0 purity). Oxygen was introduced at two locations, in the middle of the combustion tube (300 mL/min) before the enclosure with the oven and in front of the combustion tube (100 mL/min) before the sample injection port. The latter inlet was used mainly for argon, but during the final combustion, it was automatically switched to oxygen (100 mL/min). To ensure hydropyrolysis, MilliQ water was added continuously at a rate of 0.1 mL/min at two locations during the combustion process. The combustion time was controlled by a flame sensor with a 2-minute postcombustion time (minimal time in the high-temperature region). The initial absorber volume was 2 mL, and the transfer lines were rinsed with 1 mL after combustion.

To withstand the high temperatures, the combustion tube, sample boats, and the hook, to move the sample boat, were all made of quartz glass. To reduce the background fluorine signal, all polytetra-fluoroethylene (PTFE, Teflon®) tubings were replaced with tubing made of polyurethane or polyether ether ketone (PEEK). To increase the sensitivity of the system, the amount of absorber solution injected onto the IC was increased to 2 mL, for which a preconcentration column (Metrosep A PCC 2 HC/4.0) was used.

The EOF results were obtained using an external calibration curve (50 to 2500 ng/mL F). As background contamination of fluoride was observed in the CIC system, it was subtracted from samples before further data analysis. To ensure reliability, analysis of samples commenced only once the background level was stable (an RSD below 5% for the three latest background signal measurements). The CIC response was calculated by using Equation 1, which took into account the combustion blank peak area (A_c.blank_), the dilution during sample absorption (V_absorber_ and V_inj.IC_), and the initial injection volume (V_inject_). The variables are also presented in Figure S1.

Equation 1 $$CIC\;response=\left(A_{sample}-A_{c.blank}\right)\times \frac{\left(V_{absorber}÷V_{inj.IC}\right)}{V_{inject}}$$

The expected fluorine concentrations were calculated from the molecular formulas of the different PFAS; the PFAS concentrations of all analytes (ng/mL) were converted to respective fluoride concentrations (ng/mL F) using Equation 2.

Equation 2 $$C_{F}=n_{F_{i}}\times \frac{MW_{F}}{MW_{PFAS_{i}}}\times C_{PFAS_{i}}$$

where C_F_ is the fluorine equivalent concentration (ng/mL F) of the compound *i*, n_Fi_ is the number of fluorine atoms in compound *i*, MW_F_ is the molecular weight of fluorine, MW_PFASi_ is the molecular weight of compound *i*, and C_PFASi_ is the concentration of analyte *i* (ng/mL).

Calibration samples at different concentrations were compared with each other by calculating response factors (RFs), where C_prepared_ is the theoretical (prepared) concentration of the calibration sample, see Equation 3.

Equation 3 $$RF=\frac{CIC\;response}{C_{prepared}}\times 1000$$

#### Quality assurance and quality control measures

When the CIC system was initialized, it was first allowed to analyze a series of combustion blanks (empty injection in CIC, using the quartz glass boat), usually 5-10 replicates. The combustion blanks were measured using the same method as the samples to achieve a stable fluoride background level. The RSD of the three latest combustion blanks had to be below 5%, to start analyzing samples. A single combustion blank was included after every sample to evaluate possible carryover, and to track the overall condition of the system, an elevated combustion blank would have indicated contamination or other problems. The repeatability of the CIC system was estimated by analyzing a PFOA standard solution in triplicate; the RSD was 5%. The RF difference between standards prepared from solid PFOA (purchased from Fluka) and a separate premade PFOA standard solution (purchased from Wellington Labs) was 10%.

A QC solution of inorganic fluorine, analyzed through the autosampler (i.e., using combustion), was prepared from the “anion multielement standard I”, the RF difference between the QC and calibration solutions (prepared from “Fluoride standard solution”, inorganic fluorine) was 4%.

The limit of detection (LOD) was set as the lowest calibration sample for the CIC, 50 ng/mL F in the sample injected to the CIC when 0.1 mL of the sample was injected.

## Data Availability

• All data reported in this paper will be shared by the lead contact upon request. • This paper does not report original code. • Any additional information required to reanalyze the data reported in this paper is available from the lead contact upon request.
