# Supplementary material for: Combustion ion chromatography for extractable organofluorine analysis
Source: iScience. 2021 Aug 10;24(9):102968. doi: 10.1016/j.isci.2021.102968 (PMC8383001; doi:10.1016/j.isci.2021.102968)
Supplement: Document S1. Figure S1; Tables S1 and S2 [file mmc1.pdf]

iScience, Volume *24*

Supplemental information

Combustion ion chromatography for extractable organofluorine analysis

Rudolf Aro, Ulrika Eriksson, Anna Kärrman, Iris Reber, and Leo W.Y. Yeung

Supplementary Tables and Figures

(4 Pages)

Combustion Ion Chromatography for Extractable Organofluorine Analysis

Rudolf Aro[1], Ulrika Eriksson[1], Anna Kärrman[1], Iris Reber[2], Leo Wai Yin Yeung[1,3,*]

[1]Man-Technology-Environment (MTM) Research Centre, School of Science and Technology, Örebro University, Örebro, Sweden, SE-701 82
[2]Metrohm AG, Ionenstrasse, 9100 Herisau, Switzerland
[3]Lead Contact
[*]Correspondence: leo.yeung@oru.se

Table of Contents

1. Selection of Research Papers Using CIC 3
2. Method Used for CIC Analysis 4
3. CIC Scheme 4

1. Selection of Research Papers Using CIC

Table S1. Selection of studies using CIC to measure EOF. Related to Table 1.

Calibration based on		QA/QC	Matrix studied	Reference
Inorganic fluorine (IF)	NaF	N/A	water	(Miyake et al., 2007a)
	NaF	N/A	human blood	(Miyake et al., 2007b)
	NaF	10 and 100 ng/mL PFOS, 95 % and 98 % rec.[a]	human blood	(Yeung et al., 2008)
	NaF	10 and 100 ng/mL PFOS, 95 % and 98 % rec.[a]	rat blood	(Yeung et al., 2009)
	NaF	N/A	surface water, sediment, biota	(Loi et al., 2011)
	NaF	50/500 ng PFOS in serum, 80-92 % rec.[a]<br>50/500 ng PFOA, 83-90 % rec.[a]	human blood/sera	(Yeung and Mabury, 2016)
	IF solution	N/A	AFFF	(Dubocq et al., 2020)
	IF solution	N/A	river water	(Gehrenkemper et al., 2020)
	IF solution	10 and 10000 ng/mL PFBS, within +/- 10% of ref.[b] value	water	(von Abercron et al., 2019)
	NaF	BCR-461 (**IF**)	side-chain polymers	(Schellenberger et al., 2019)
	IF	N/A	surface and ground water	(Wagner et al., 2013)
Organofluorine (OF)	PFOS	250 ng PFOS spike, 73 % rec.[a]	human blood	(Miaz et al., 2020)
	PFOS	PFOS spike 7.6 ug, rec.[a] 69%<br>IF CRM (BCR-461) in agreement	cosmetics	(Schultes et al., 2018)
	PFOA	N/A	IDW pilot	(Singh et al., 2019)
	PFOS	PFOS	cod	(Schultes et al., 2020b)
	PFOS	PFOS and BCR-461	killer whale	(Schultes et al., 2020a)

[a]rec: recovery
[b]ref: reference

2. Method Used for CIC Analysis

Table S2. Parameters of the CIC Method. Related to STAR methods.

Parameter	Value		Parameter	Value		Parameter	Value
Injection volume from the sample	100 µL		Minimal time at the combustion zone[a]	2 min		Trap column used on the IC	Metrosep A PCC 2 HC/4.0
Boat material	Quartz		Absorber solution	H_2O		Analytical column on the IC	Metrosep A Supp 5 – 150/4.0
Pyrolysis gas	Argon (6.0)		Initial absorber solution volume	2 mL		Mobile phase	64 mmol/L Na_2CO_3 and 20 mmol/L $NaHCO_3$
Pyrolysis gas flow speed	100 mL/min		Rate of absorber solution addition[b]	0.1 mL/min		Mobile phase flow rate	0.7 mL/min
Combustion gas	Oxygen (5.0)		Amount of water used to rinse transfer lines after combustion	1 mL		Column temperature	40 °C
Combustion gas flow speed	300 mL/min		Amount of water injected on IC	2 mL			
Oven temperature	1050 °C						

[a]End of the combustion tube where the temperature is the highest.

[b]Water was added during combustion at two points, see Figure S1.

3. CIC Scheme

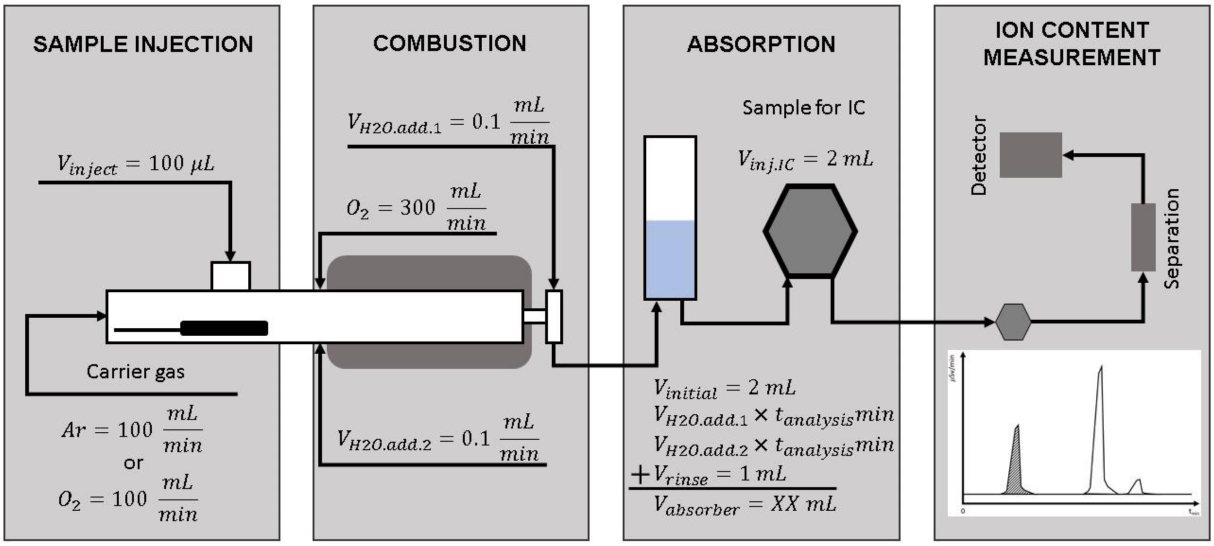

Figure S1. Schematic representation of the CIC system. Related to STAR methods.
